# Boundary‐Dependent Sleep–Wake Dysregulation in Idiopathic Hypersomnia

**DOI:** 10.1002/acn3.70522

**Published:** 2026-09-02

**Authors:** Samantha Mombelli, Karine Lacourse, Hélène Blais, Anne‐Sophie Deshaies‐Rugama, Cynthia Thompson, Alex Desautels, Jacques Montplaisir, Milan Nigam, Christophe Moderie, Boshra Khajehpiri, Catherine Duclos, Jean Marc Lina, Julie Carrier, Nadia Gosselin

**Affiliations:** ^1^ Center for Advanced Research in Sleep Medicine Research Center of the Centre intégré Universitaire de Santé et de Services Sociaux du Nord‐de‐l'Île‐de‐Montréal Montreal Canada; ^2^ Department of Psychiatry and Addictology Université de Montréal Montreal Canada; ^3^ Department of Psychology Université de Montréal Montreal Canada; ^4^ Department of Neuroscience Université de Montréal Montreal Canada; ^5^ LIVIA, ILLS, École de technologie supérieure (ÉTS) Université du Québec Montreal Canada; ^6^ Department of Electrical Engineering École de technologie supérieure Montreal Quebec Canada; ^7^ Department of Anesthesiology and Pain Medicine Université de Montréal Montreal Canada

**Keywords:** non‐restorative sleep, sleep microstructure, sleep–wake balance, sleepiness, thalamocortical network

## Abstract

**Objective:**

Idiopathic hypersomnia (IH) presents with excessive daytime sleepiness (EDS) despite apparently preserved nocturnal sleep, challenging traditional models of hypersomnolence based on sleep loss or fragmentation. We aimed to test the hypothesis that EDS in IH reflects excessive stabilization of the sleep state, consistent with dysfunctional thalamocortical control rather than impaired sleep quantity or continuity.

**Methods:**

We analyzed overnight polysomnography from 62 IH and 81 age‐ and sex‐matched healthy controls using sleep bout duration, sleep stage transition dynamics, and automated spindle detection. Principal component analyses derived composite indices of NREM and REM sleep stability. Group differences were assessed using ANCOVAs controlling for age and sex, and associations with EDS severity were examined.

**Results:**

Compared with controls, IH patients showed reduced transitions from N3 sleep toward lighter stages and wakefulness. Moreover, REM sleep was characterized by longer durations, fewer transitions to wakefulness, and more frequent transitions to lighter stages. In parallel, spindle amplitude was reduced in IH while spindle density was preserved, and smaller spindle amplitudes were associated with shorter latencies on the Multiple Sleep Latency Test.

**Interpretation:**

IH is characterized by excessive N3 stabilization and reduction in transitions toward wakefulness in both NREM and REM sleep. These findings suggest that altered sleep–wake dynamics, rather than impaired sleep continuity, may contribute to persistent daytime sleepiness in IH and support sleep stability as a potential target for future research and therapeutic strategies.

## 1 Introduction

Excessive daytime sleepiness (EDS) is the hallmark symptom of idiopathic hypersomnia (IH), a central disorder of hypersomnolence often accompanied by prolonged sleep duration [1]. Despite preserved macrostructural sleep architecture [2, 3, 4], patients report unrefreshing sleep and morning inertia [5, 6]. This paradox challenges models linking sleepiness to fragmented or insufficient sleep [7, 8], suggesting that the pathophysiology of IH may lie in how the brain stabilizes and transitions between vigilance states.

Sleep–wake regulation depends on a delicate balance between sleep stability and reversibility, defined as the ability to appropriately transition from sleep to wakefulness in response to internal signals (e.g., circadian arousal drives) and external demands. Insufficient stability leads to fragmented and non‐restorative sleep, whereas excessive stability may result in a rigid sleep state that is poorly responsive to homeostatic, circadian, or environmental demands. From this perspective, IH represents a paradoxical phenotype. Patients show a reduced ability to sustain prolonged wakefulness, as evidenced by EDS and impaired performance on the wakefulness test [9, 10], despite normal or supranormal nocturnal sleep efficiency [3, 11]. This dissociation supports the idea that IH may involve an imbalance in sleep–wake regulation with an excessively stable sleep state.

One way to capture this regulatory balance is to examine the internal organization of sleep, including the duration of uninterrupted REM and NREM bouts and the dynamics of transitions between vigilance states. Longer sleep bouts reflect the capacity to sustain consolidated sleep (stability), whereas the frequency and direction of sleep stage transitions capture how the system reorganizes across vigilance states and transitions toward wakefulness (reversibility). Previous PSG studies suggest that IH is characterized by longer N2 bouts and a global reduction in sleep‐stage transitions, particularly from N3 toward lighter stages [12, 13]. However, these findings are based on small and/or heterogeneous samples, including pediatric populations, with samples of 14 and 18 individuals with IH, respectively.

At the microstructural level, sleep spindles, transient oscillations generated by thalamocortical circuits during NREM sleep [14, 15], play a key role in sensory gating and sleep stability by reducing cortical responsiveness to external stimuli [16]. In good sleepers, spindles reflect adaptive thalamocortical engagement to protect sleep [17, 18]. Two studies reported higher spindle density in IH (11 and 8 participants, respectively) [19, 20], suggesting enhanced sleep‐stabilizing mechanisms. However, whether spindle characteristics in IH reflect compensatory mechanisms or maladaptive thalamocortical over‐stabilization remains unclear, particularly in the absence of detailed morphological analyses and adequately powered samples.

Joint examination of sleep bout dynamics, stage transitions, and spindle characteristics offers a window onto the balance between sleep‐promoting and arousal‐promoting networks involving thalamic, hypothalamic, and brainstem circuits [21, 22]. When this balance is pathologically skewed toward hyperstability, sleep becomes excessively prolonged and poorly reversible [23]. Conversely, reduced stability leads to fragile and fragmented sleep, as observed in insomnia [24, 25], narcolepsy [26], and other sleep disorders [27, 28].

In this study, we aimed to characterize the electrophysiological balance between NREM and REM sleep stability in IH by integrating sleep bout duration, transition dynamics, and spindle morphology. We hypothesized that IH would be characterized by longer N2–N3 and REM bouts, fewer transitions toward wakefulness, and altered spindle features reflecting excessive sleep stability. We further hypothesized that greater electrophysiological stability would correlate with higher EDS, capturing the behavioral consequences of altered sleep–wake transition dynamics.

## 2 Materials and Methods

### 2.1 Participants and Overview of the Protocol

**FIGURE 1:**
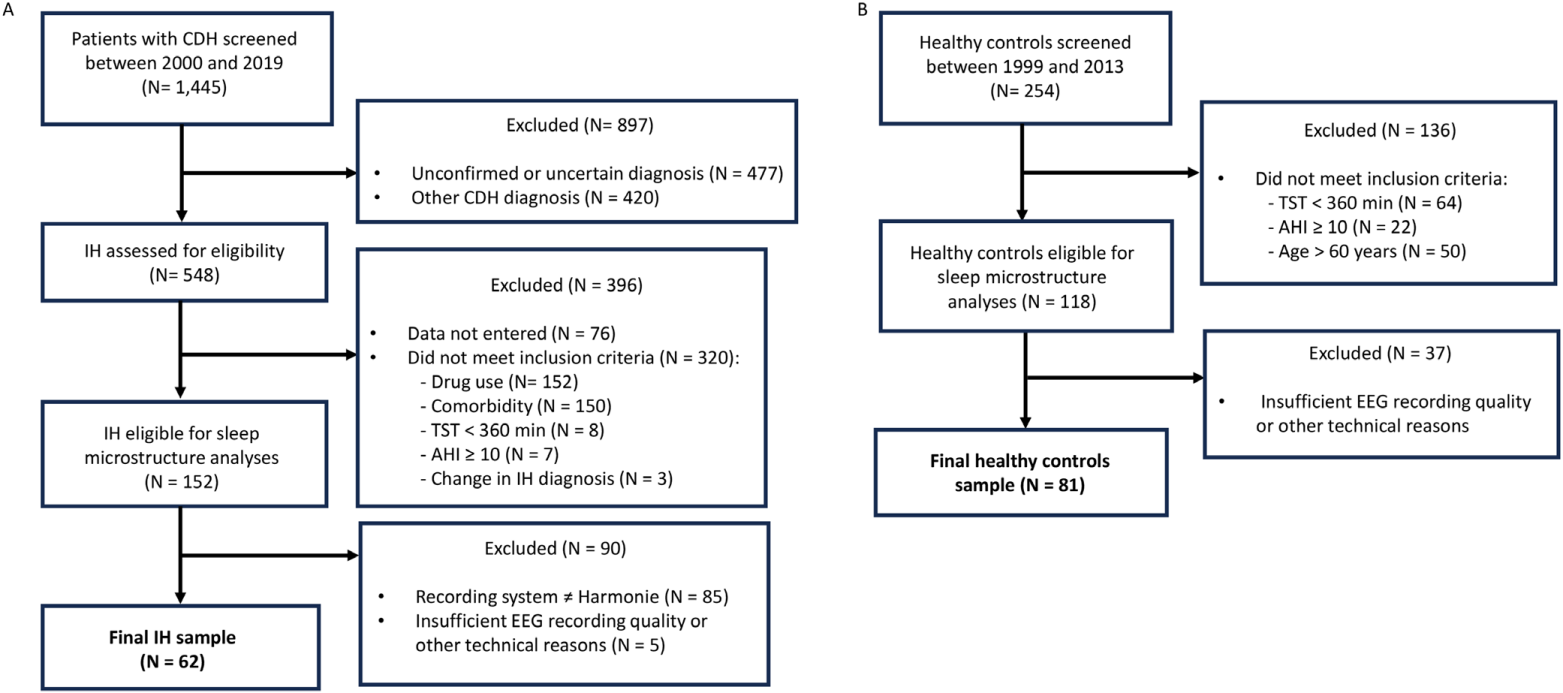
Flow charts of sample selection for IH patients and healthy controls. (A) The patient flow chart reflects the study‐specific selection criteria applied to the current dataset. The resulting sample is therefore not intended to be representative of, or directly comparable with, samples from previous studies derived from the same cohort, which relied on different selection procedures and variable availability. (B) Healthy controls were drawn from multiple pre‐existing research protocols included in the CARSM Nights Bank (REB 2020–1978). Consequently, some inclusion and exclusion criteria relevant to the present study had already been implemented during prior data collection. The control flow chart therefore reflects only the selection steps applied within the framework of the current study. CDH, central disorders of hypersomnolence; IH, idiopathic hypersomnia.

IH patients assessed at the Center for Advanced Research in Sleep Medicine (CARSM) clinic, located at the Hôpital du Sacré‐Cœur de Montréal, Montreal, Canada, between 2000 and 2019 were retrospectively screened for eligibility. Participants in the IH group were selected based on the availability of an overnight in‐laboratory PSG and an MSLT. The following inclusion criteria were also applied: (1) aged between 18 and 60 years at the time of testing; (2) diagnosis of IH based on ICSD 3‐TR criteria [1]; (3) a minimum of 6 h of sleep recorded during PSG; (4) a mean sleep onset latency ≤ 8 min on the MSLT; and (5) fewer than two sleep onsets in REM periods (SOREMP) across the PSG‐MSLT procedure. Exclusion criteria included: (1) diagnostic revision over time, defined as any revision of the initial IH diagnosis on follow‐up visits; (2) apnea hypopnea index (AHI) ≥ 10; (3) psychiatric or neurological disorder, or another sleep disorder; (4) shift work; (5) circadian rhythm disorder or chronic sleep deprivation; and (6) use of medications known to affect sleep (antidepressants, psychostimulants, antipsychotics, anxiolytics, hypnotics, opioids) that were not discontinued for at least five half‐lives prior to the PSG. At the sleep clinic, each patient underwent a clinical interview conducted by a sleep medicine specialist. This interview gathered demographic and clinical information relevant to IH. Participants also completed the Epworth Sleepiness Scale (ESS) to assess subjective daytime sleepiness [29]. A cohort of healthy control participants, aged 18–60 years, was assembled from the CARSM Nights Bank (REB 2020–1978). All underwent sleep assessments at the CARSM between 1999 and 2013. As part of the study‐specific retrospective selection procedure, healthy controls were required to have a minimum of 6 h of sleep recorded during the overnight PSG. Exclusion criteria were otherwise identical to those applied to the IH group. The final IH sample consisted of 62 individuals with IH and 81 healthy controls (Figure 1). The study was conducted in accordance with the Declaration of Helsinki and approved by the local Research Ethics Board of the CIUSSS du Nord‐de‐l'Île‐de‐Montréal (REB 2020–1905).

### 2.2 Polysomnography Recording and MSLT

All participants underwent overnight in‐laboratory PSG scheduled according to their habitual sleep–wake patterns (typically with lights‐off between 10:00 PM and 11:00 PM, and wake time no later than 7:00 AM). The PSG recordings included a minimum 4‐channel EEG montage (C3, C4, O1, and O2) following the international 10–20 system, with signals referenced to linked mastoids. PSG also included bilateral electrooculography, submental and bilateral anterior tibialis electromyography, and electrocardiography. Respiration was measured through a thoracoabdominal strain gauge, oronasal cannula, and thermistors, while oxygen saturation was measured through a finger pulse oximeter. All signals were digitized at a sampling rate of 256 Hz using Harmonie software (Stellate Systems, Montreal, Canada). Sleep staging and event scoring were performed manually by an experienced sleep technologist in accordance with the more recent criteria established by the American Academy of Sleep Medicine [30]. The following sleep variables were derived: total sleep time, wake after sleep onset, number of awakenings, sleep onset latency, sleep efficiency, percentage of sleep stages (N1, N2, N3, REM), and AHI. Apneas were defined as a ≥ 90% reduction in airflow lasting ≥ 10 s, while hypopneas were defined as a ≥ 30% airflow reduction for at least 10 s, associated with either a ≥ 3% oxygen desaturation or an arousal [30]. For patients with IH, an adapted version of the MSLT was performed the following day of PSG, consisting of four nap opportunities scheduled at 09:00, 11:00, 13:00, and 15:00. SOREMPs were defined as the occurrence of REM sleep within 15 min of sleep onset, either during the overnight PSG or any of the MSLT naps. Mean sleep onset latency across the four naps was calculated for each participant.

### 2.3 Sleep Bout Duration and Stage Transitions

Sleep bouts were identified across the entire night using the Snooz Toolbox version beta‐2.0.0, an open‐access platform developed by our group (snooztoolbox.com), which segments uninterrupted periods of sleep according to predefined stage groupings. For N2–N3 bouts, continuous sequences of N2 and N3 epochs were considered part of the same bout, regardless of transitions between these two stages. A bout was terminated only by the occurrence of wake or a sleep stage outside the N2–N3 category. For REM bouts, continuity was defined as uninterrupted REM sleep, and the bout ended at the first occurrence of wake or any non‐REM stage, as previously described [31]. Bout duration was computed in minutes for each continuous episode within a given sleep stage category, with separate analyses for N2–N3 and REM sequences. For each participant, we extracted the duration of the 10 longest bouts per sleep category (N2–N3, REM), as well as their mean duration and standard deviation. Additionally, averaged durations and standard deviations were calculated for all bouts in each category. Bout duration variables were analyzed as absolute measures and were not normalized to total sleep time or time in bed, as they were intended to capture the persistence of a given sleep state independently of overall sleep duration. We also measured the transition index (number of sleep stage transitions/h) from one stage (awake, N1, N2, N3, and REM) to any other stage throughout the night, and a global sleep stage transition index (total number of transitions/recording hours).

### 2.4 Spindle Detection

Artifacts were identified using automatic homemade software (HarmAct) and subsequently reviewed by senior sleep technologists. EEG recordings were visually inspected to validate identified artifacts and detect additional artifact‐contaminated segments. Artifact classes included flatline segments (low‐power or flatlined signal), high‐frequency bursts, ECG contamination, persistent high‐frequency noise, power‐line contamination (60 Hz), baseline variations associated with breathing, and muscle artifacts. Channels that were heavily contaminated throughout the recording were excluded from spindle analyses. Artifact‐contaminated signal segments were masked (minimum duration 0.5 s), preventing spindle detection within those intervals while preserving the remaining valid EEG data. Entire N2/N3 epochs were excluded only when completely corrupted by artifacts. Sleep spindles were automatically detected on central (C3–C4) derivations during N2 and N3 sleep across the entire night using the SUMO detector [32], a neural network–based algorithm implemented in the Snooz Toolbox (version beta‐2.0.0). The SUMO (Slim U‐Net trained on MODA) algorithm is a deep learning model based on the U‐Net architecture, trained to approximate expert consensus annotations from the Massive Online Data Annotation (MODA) project, which aggregates multiple human scorers to improve reliability [33]. Unlike traditional feature‐based detectors, SUMO uses deep convolutional networks to process minimally preprocessed EEG signals, enhancing generalizability and reducing dependence on handcrafted features. For each participant, spindle density (number of events per minute of N2–N3 sleep), duration, mean frequency based on peak counting (Hz), mean peak‐to‐peak amplitude (μV), and mean Root Mean Square (RMS) amplitude (μV) were extracted. Values from C3 and C4 were averaged prior to statistical analysis.

### 2.5 Statistical Analyses

Analyses were conducted on complete cases only, and no data imputation was performed. Group differences (IH vs. controls) in demographic, clinical, and sleep macroarchitecture variables were assessed using independent‐samples *t*‐tests, Mann–Whitney U tests, or chi‐square tests, as appropriate. Principal component analysis (PCA) was performed on bout‐ and transition‐related variables across the whole sleep recording. For each participant, N2–N3 and REM stability were indexed by the mean and standard deviation of all and of the 10 longest uninterrupted bouts, while transition dynamics were represented by the normalized frequency (number/h) of stage‐to‐stage (Wake, N1, N2, N3, and REM) transitions. Spindle characteristics and PCA‐derived indices were compared between IH and control groups using one‐way ANCOVAs with age and sex as covariates. Within the IH group, partial correlations controlling for age and sex were conducted between markers of EDS (ESS scores and mean sleep onset latency on the MSLT) and spindle or PCA parameters that significantly differed between groups. For all statistical tests, we performed the Benjamini–Hochberg false‐discovery rate procedure for multiple comparisons. All analyses were conducted using JASP software (version 0.19).

## 3 Results

### 3.1 Participant Characteristics

Table 1 presents demographic, clinical, and PSG results for IH and control groups. Compared to the control group, the IH group showed significantly longer total sleep time, higher sleep efficiency, reduced wake after sleep onset, and fewer awakenings during the nighttime PSG. Moreover, IH participants spent more time in N2 and REM sleep stages compared to the control group.

**TABLE 1:** Demographic, clinical, and polysomnographic data of IH and control groups.

	IH *n* = 62	Controls *n* = 81	Statistics^a^	*p*‐value*	Effect sizes
**Demographic/clinical data**					
Age, years	35.34 ± 10.48	35.27 ± 15.20	2332.50	0.468	−0.071
Women, *n* (%)	43 (69.4%)	45 (55.6%)	2.83	0.093	0.141
Body mass index, kg/m^2^	24.75 ± 3.88	24.01 ± 3.85	2194.00	0.240	−0.115
Epworth Sleepiness Scale scores	15.26 ± 4.34	NA	NA	NA	NA
Mean sleep onset latency on MSLT, min	4.93 ± 1.88	NA	NA	NA	NA
**PSG parameters**					
Total sleep time, min	460.15 ± 28.79	418.51 ± 47.77	1035.50	**0.005**	−0.588
Sleep onset latency, min	9.36 ± 8.53	16.75 ± 24.95	3000.00	0.118	0.195
REM latency, min	91.03 ± 36.98	107.76 ± 52.99	2909.00	0.105	0.159
Wake after sleep onset, min	30.21 ± 21.32	43.49 ± 30.31	3166.00	**0.005**	0.261
Awakenings, n	21.40 ± 9.16	26.73 ± 11.54	3194.00	**0.014**	0.272
Sleep efficiency, %	93.81 ± 4.25	90.59 ± 6.32	1725.00	**0.005**	−0.313
N1 sleep duration, min	44.50 ± 20.35	39.35 ± 20.29	2873.00	0.141	−0.144
N2 sleep duration, min	254.25 ± 38.19	232.75 ± 40.69	3.215	**0.007**	0.542
N3 sleep duration, min	66.26 ± 36.26	65.12 ± 39.59	0.177	0.860	0.030
REM sleep duration, min	95.87 ± 26.82	81.29 ± 23.10	3277.00	**0.007**	−0.305
N1 sleep proportion, %	9.75 ± 4.77	9.42 ± 4.77	2474.50	0.883	−0.015
N2 sleep proportion, %	55.21 ± 7.76	55.64 ± 7.28	0.34	0.734	0.057
N3 sleep proportion, %	14.37 ± 7.87	15.49 ± 9.20	2607.50	0.696	0.038
REM sleep proportion, %	20.69 ± 5.25	19.46 ± 5.02	−1.42	0.157	−0.240
Micro‐arousal index, n/h	9.19 ± 5.67	8.56 ± 4.53	2372.00	0.464	−0.124
Apnea‐hypopnea index, n/h	2.41 ± 3.16	1.70 ± 2.43	2047.50	0.059	−0.185
Periodic limb movements index, n/h	7.07 ± 8.41	5.93 ± 9.91	2057.00	0.064	−0.181

*Note:* Data are presented as mean ± standard deviation for continuous variables, and as the number of participants and percentage for categorical variables. Bold *p*‐values indicate statistically significant results (*p* < 0.05).

Abbreviations: IH, idiopathic hypersomnia; MSLT, multiple sleep latency test; PSG, polysomnography; REM, rapid eye movement.

* *p*‐values are corrected for multiple comparisons.

^a^ Statistics refer to Student's *t*‐test, Welch's *t*‐test, or Mann–Whitney *U* values for continuous variables, depending on the distribution of the data and the homogeneity of variances. For categorical variables, χ^2^ values are reported. ^b^Effect sizes are reported as Hedges' *g* for continuous variables and Cramer's *V* for categorical variables.

### 3.2 Principal Component Analysis

PCA was intentionally designed as a physiological synthesis rather than a latent‐factor model, retaining heterogeneous but conceptually related sleep variables to capture complementary aspects of sleep stability. We distinguished “within‐sleep stability”, referring to transitions between sleep stages, from “sleep–wake boundary stability”, referring to transitions between sleep and wakefulness. Preliminary tests supported the suitability of the data for factor extraction: Bartlett's test of sphericity was significant (χ^2^ (226) = 4584.061, *p* < 0.001), indicating sufficient correlations among variables, although the overall Kaiser–Meyer–Olkin value of 0.39 suggested modest shared variance, consistent with the heterogeneity of sleep‐stage metrics. PCA with Varimax rotation identified five components (eigenvalues > 1) explaining 66.6% of total variance; scree plot and parallel analysis supported retention of this solution (see Table 2, Figure 1‐S1, and Table 1‐S1 in Supporting Information S1).

**TABLE 2:** PCA component loadings.

Variables	PC1 NREM sleep instability	PC2 within‐REM sleep stability	PC3 N3 sleep instability	PC4 REM‐N2 instability	PC5 REM‐wake instability	Uniqueness
SD of all uninterrupted N2−N3 bouts	−0.930					0.126
Mean of 10 longest N2−N3 bouts	−0.893					0.186
Transition index from N1 to N2 sleep	0.874					0.143
Mean of all uninterrupted N2−N3 bouts	−0.840					0.221
Transition index from Wake to N1	0.794					0.286
Transition index from N2 to N1	0.729					0.287
Transition index from N2 to Wake	0.716					0.336
SD of 10 longest N2−N3 bouts	−0.700					0.484
Transition index from N1 to Wake	0.637					0.548
SD of all uninterrupted REM bouts		0.873				0.162
Mean of all uninterrupted REM bouts		0.839				0.130
SD of 10 longest REM bouts		0.835				0.236
Transition index from N1 to REM		−0.765				0.317
Transition index from REM to N1		−0.764				0.367
Mean of 10 longest REM bouts		0.656				0.403
Transition index from N2 to N3			0.776			0.310
Transition index from N3 to Wake			0.761			0.416
Transition index from N3 to N2			0.712			0.387
Transition index from N1 to N3			0.702			0.460
Transition index from N3 to N1			0.553			0.418
Transition index from Wake to N3			0.541			0.652
Transition index from REM to N2				0.907		0.146
Transition index from N2 to REM				0.875		0.160
Transition index from REM to Wake					0.771	0.224
Transition index from Wake to REM					0.748	0.335
Transition index from Wake to N2						0.581
Transition index from N3 to REM						0.699

*Note:* The applied rotation method is Varimax.

Abbreviations: NREM, non–rapid eye movement; PC, principal component; REM, rapid eye movement; SD, standard deviation.

The first component (PC1) reflected both within NREM instability and NREM‐wake boundary instability, with negative loadings on the duration and variability of uninterrupted N2–N3 bouts and positive loadings on transitions from NREM to lighter sleep stages or wakefulness. Higher PC1 scores indicated greater NREM instability.

The second component (PC2) represented within‐REM sleep stability, with positive loadings on REM bout metrics and negative loadings on transitions from REM to N1, indexing REM fragmentation. Higher PC2 scores correspond to more stable REM sleep.

The third component (PC3) captured N3 sleep instability with strong positive loadings on cross‐stage transitions involving N3 and wake or other NREM stages. Higher PC3 scores indicate higher instability.

While PC2 captured within‐REM sleep stability, the remaining components refined REM regulation by focusing on transitions involving specific states. The fourth component (PC4) represented within‐sleep instability involving REM and N2, characterized by strong positive loadings on bidirectional transitions between these stages. Higher PC4 scores indicated more frequent REM–N2 transitions and greater instability between these sleep stages.

The fifth component (PC5) captured instability at the REM–wake boundary, with positive loadings on bidirectional transitions between REM sleep and wakefulness. Higher PC5 scores indicated more frequent REM–wake transitions and greater sleep–wake boundary instability.

### 3.3 Group Comparisons on Sleep Bout Duration and Stage Transition Components

Group results and comparisons for individual sleep bout duration and sleep stage transition variables are presented in Supporting Information S2 (see Table 1‐S2 and 2‐S2). Globally, the IH group exhibited longer REM sleep bouts, specifically among the 4th to the 10th longest uninterrupted REM episodes. Regarding transition dynamics, IH participants showed fewer transitions from wakefulness to N1 and N2, as well as fewer transitions from N2 and N3 toward wakefulness and between N2 and N3. In contrast, they exhibited increased transitions from N2 to N1 and between REM and N1 sleep.

**FIGURE 2:**
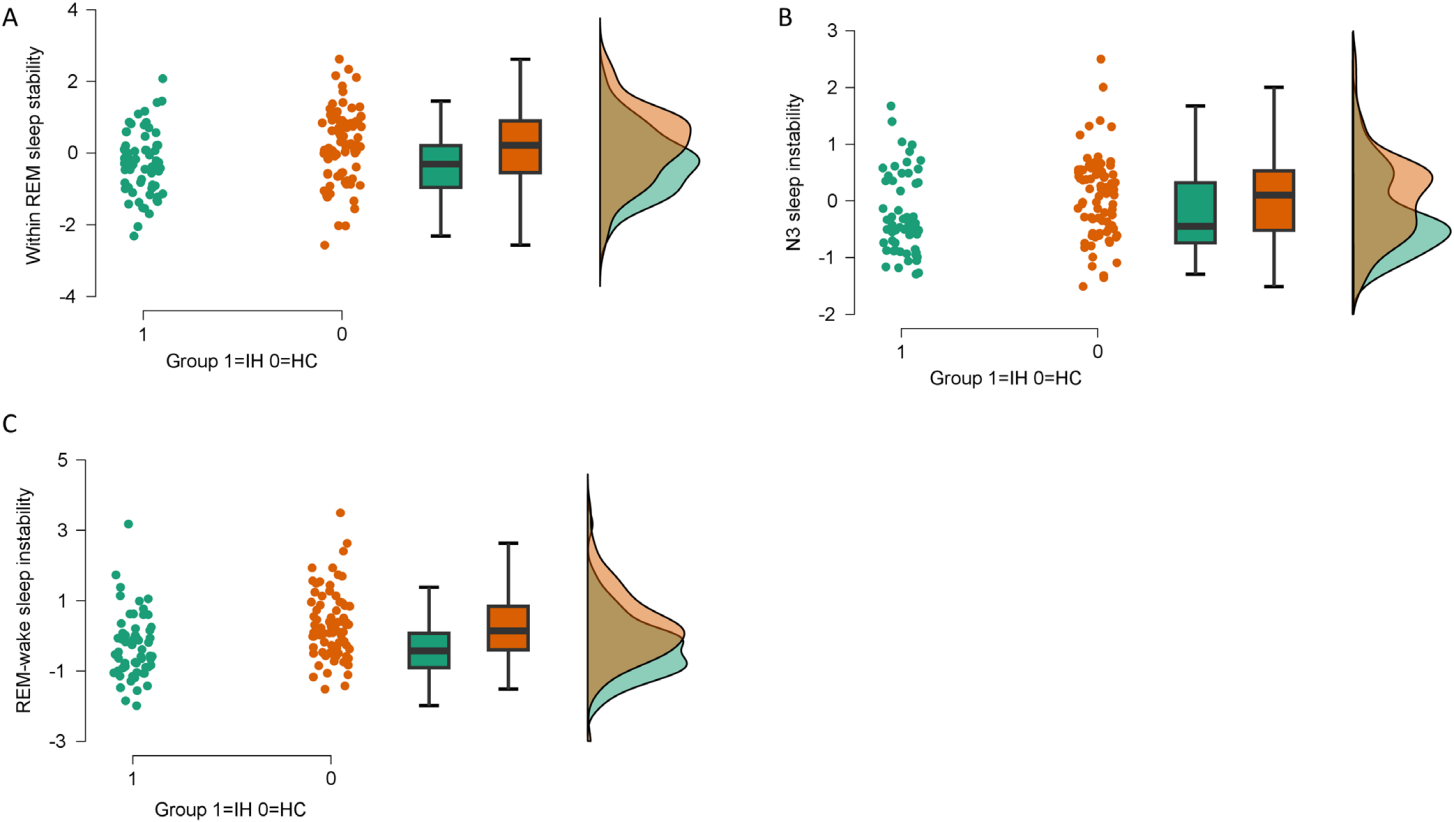
Group differences in PCA‐derived indices representing sleep stability. Individual data points (left) represent participant‐level values, box plots (center) indicate medians and interquartile ranges, and density plots (right) illustrate score distributions for idiopathic hypersomnia (IH, green) and healthy controls (orange). (A) Distribution of principal component (PC2) scores indexing within‐REM sleep stability. Higher scores indicate greater stability and lower fragmentation. (B) Distribution of principal component (PC3) scores indexing N3 sleep instability. Lower scores indicate greater stability with less frequent transitions involving N3 sleep. (C) Distribution of principal component (PC5) scores indexing REM‐wake sleep instability. Lower scores indicate greater stability at the REM‐wake boundary, with less frequent bidirectional transitions between REM sleep and wakefulness. IH, idiopathic hypersomnia; HC, healthy controls; NREM, non–rapid eye movement; REM, rapid eye movement; PC, principal component.

Compared with controls, IH participants showed lower within‐REM sleep stability on PC2 (*F* (1,139) = 11.631, *p* = 0.003 corrected), increased N3 stability on PC3 (*F* (1,139) = 8.804, *p* = 0.006 corrected), and less REM‐wake instability on PC5 (*F* (1,139) = 10.961, *p* = 0.003 corrected). For PC3, exclusion of the three control participants identified as potential outliers yielded similar effect estimates, and the group difference remained statistically significant (corrected *p* = 0.009). No significant difference emerged for PC1 and PC4. Figure 2 illustrates significant group results.

### 3.4 Sleep Spindles in IH Compared to Controls

Table 3 presents data and statistics for spindles detected across N2 and N3 sleep at central derivations (C3–C4). Spindle amplitudes (peak‐to‐peak and RMS) were significantly lower in the IH group compared to controls. No significant group differences were observed for spindle density and duration. However, spindle frequency showed a non‐significant trend toward higher values in the IH group compared with controls.

**TABLE 3:** All‐night spindle characteristics across N2 and N3 sleep at central derivations (C3–C4) in IH and control groups, detected using the SUMO algorithm and adjusted for age and sex.

	IH *n* = 62	Controls *n* = 81	*F* value (1, 139)	*p*‐value*	Effect sizes
Density, n/min	2.90 ± 1.77	3.11 ± 1.81	0.575	0.450	0.004
Duration, s	0.85 ± 0.10	0.86 ± 0.10	1.284	0.259	0.007
Spindle average frequency counting peaks, Hz	13.52 ± 0.43	13.35 ± 0.43	3.088	0.081	0.019
Peak‐to‐peak amplitude, μV	31.80 ± 6.77	33.53 ± 6.41	6.652	**0.027**	0.035
Root mean square amplitude, μV	8.20 ± 1.71	8.71 ± 1.64	7.647	**0.027**	0.040

*Note:* Data are presented as mean ± standard deviation. Bold *p*‐values indicate statistically significant results (*p* < 0.05).

Abbreviations: C3–C4, central EEG derivations; Hz, hertz; IH, idiopathic hypersomnia; μV, microvolts; s, seconds.

* *p*‐values are corrected for multiple comparisons.

### 3.5 Correlations With Excessive Daytime Sleepiness Among the IH Group

**FIGURE 3:**
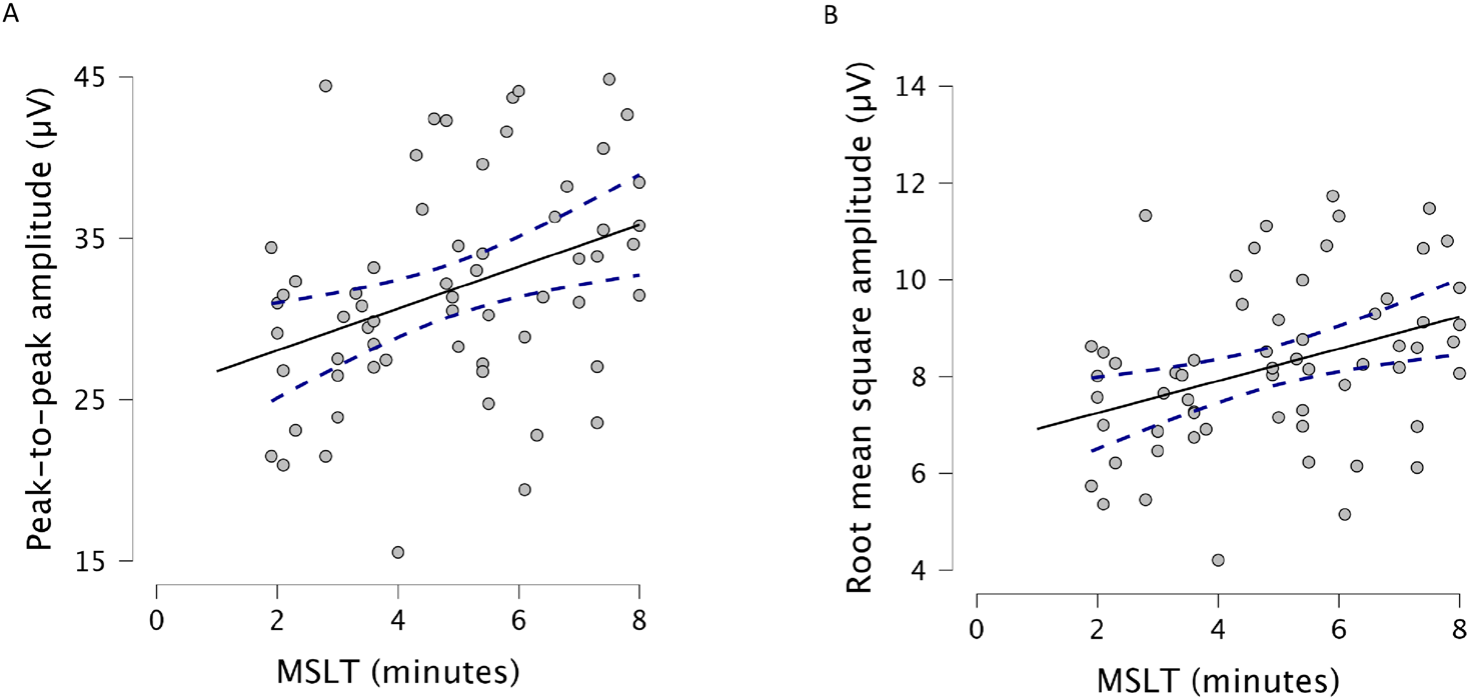
Associations between spindle amplitudes and MSLT in IH patients. Scatter plots illustrate the associations between objective daytime sleepiness, indexed by mean sleep onset latency on the MSLT, and spindle amplitudes in IH. Each dot represents one participant, and regression lines indicate the direction of the relationship. (A) Peak‐to‐peak amplitude (μV): Higher MSLT latencies (indicating lower sleepiness) were associated with greater spindle amplitudes. (B) Root Mean Square (RMS) amplitude (μV): A similar positive association was observed, suggesting that reduced daytime sleepiness parallels stronger spindle expression during N2‐N3 sleep. *p‐values are corrected for multiple comparisons. MSLT, Multiple Sleep Latency Test; μV, microvolts.

Correlations were only tested for variables where Group effects were found. Shorter mean sleep latency on MSLT was correlated with smaller spindle peak‐to‐peak amplitude (*r* = 0.296, *p* = 0.022) (Figure 3A) and smaller root mean square amplitude (*r* = 0.306, *p* = 0.017) (Figure 3B). No associations emerged between ESS scores and spindle features. Moreover, none of the three main PCA components correlated with either MSLT sleep onset latency or ESS.

### 3.6 Sensitivity Analyses

Given that sleep spindles are most prominently expressed during N2 sleep, we conducted sensitivity analyses comparing the two groups on spindle‐related variables restricted to N2 sleep (see Table 1‐S3 in Supporting Information S3). These analyses yielded results consistent with those obtained in the primary analysis, including both N2 and N3 sleep. Additional channel‐specific sensitivity analyses were conducted separately for C3 and C4, using the same age‐ and sex‐adjusted models, to assess whether the averaged C3–C4 results could mask potential lateralized effects. The pattern of findings was consistent across both derivations, with reduced spindle amplitudes in IH observed at both C3 and C4, indicating that the main spindle results were not driven by a single channel.

## 4 Discussion

The present study identifies a sleep profile in IH characterized by altered REM dynamics and excessive stability of N3 sleep. REM sleep showed reduced within‐sleep stage stability, reflected by more frequent REM‐N1 transitions despite longer REM duration. However, it showed increased REM‐wake stability, reflected by fewer transitions from REM sleep to wakefulness. Rather than a globally unstable REM state, these findings suggest a redistribution of stability across boundaries, with transitions preferentially maintained within the sleep domain rather than toward wakefulness. Conversely, increased N3 stability may reflect either heightened sleep drive or reduced responsiveness of deep sleep to internal and external demands, with fewer transitions toward lighter stages or wakefulness. This rigidity is consistent with an over‐engagement of sleep‐promoting mechanisms that sustain continuity at the expense of adaptability. IH participants also showed reduced sleep spindle amplitudes but preserved density when compared to healthy controls, and smaller spindle amplitudes correlated with shorter sleep latency on the MSLT, suggesting reduced thalamocortical engagement in a globally hyperstable NREM sleep system. Together, this sleep‐dynamics profile indicates a dysregulation of sleep–wake control that may underlie the paradox of apparently efficient yet non‐restorative sleep and contribute to the EDS characteristic of IH.

### 4.1 Boundary‐Dependent REM Sleep Stability in IH

Despite total time spent in REM sleep being prolonged in IH, REM sleep dynamics reveal a paradoxical and boundary‐dependent pattern of regulation. Other studies have shown that patients with IH spend more time in REM sleep [2], and may exhibit longer uninterrupted REM bouts [12]. However, the present results indicate that REM sleep in IH is not uniformly stabilized across all transitions, but instead characterized by increased within‐REM sleep instability, reflected by more frequent transitions between REM and N1 sleep compared to controls. This fragmentation suggests that the circuits coordinating REM onset and offset, principally GABAergic neuron populations within the sublaterodorsal tegmental nucleus and the ventrolateral periaqueductal gray/lateral pontine tegmentum [22], may operate within a narrow and unstable boundary zone [34]. Importantly, this within‐REM sleep instability coexists with increased stability at the REM‐wake boundary, indicating that REM sleep is less likely to transition into wakefulness once entered. Together, these findings indicate that REM sleep in IH is fragmented within the sleep domain, yet relatively protected from transitions to wakefulness, resulting in increased REM sleep expression despite within‐REM sleep instability. This pattern contrasts with the pervasive and abrupt REM intrusions typical of narcolepsy type 1, where orexin deficiency destabilizes the entire sleep–wake boundary, leading to frequent REM intrusions into wakefulness, SOREMPs, cataplexy, and other REM‐related phenomena [35, 36, 37]. In IH, this configuration is consistent with a hyperstable sleep–wake regulatory system characterized by reduced permeability at the sleep–wake boundary and sustained confinement within sleep states. In line with this interpretation, all IH participants exhibited fewer than two SOREMPs on the MSLT, had no cataplexy, and none experienced diagnostic changes since their initial evaluation. Moreover, REM‐related PCA components were not associated with objective or subjective measures of EDS, suggesting that the dissociation between within‐REM sleep instability and REM‐wake stability reflects a specific alteration of sleep‐state regulation rather than a direct driver of EDS.

### 4.2 Increased N3 Sleep Stability in IH

The present study also reveals that IH participants exhibited fewer transitions from N3 sleep toward wakefulness or lighter sleep stages, indicating that once deep sleep is established, it tends to persist more than in healthy controls. During NREM sleep, these transitions dynamically regulate sleep depth and facilitate the progressive reactivation of cortical and subcortical arousal systems in preparation for awakening [38, 39]. Their reduction in IH therefore points to an excessive stabilization of thalamocortical networks that impairs sleep flexibility and may hamper the ability to upregulate cortical activation when approaching wakefulness [40]. Alternatively, this deep‐sleep persistence could reflect an abnormally sustained homeostatic drive, with slower dissipation of sleep pressure maintaining cortical synchronization and delaying the normal reorganization of sleep stages across the night [41]. Such over‐stabilization of N3 may paradoxically trap the system in non‐adaptive states that resist transition to full alertness. Inefficient recruitment of wake‐promoting networks could further reinforce this inertia, giving rise to the clinical manifestations of excessive daytime sleepiness and sleep drunkenness [6]. However, excessive N3 stability did not correlate with subjective or objective measures of EDS, indicating that these dynamics may contribute to the overall physiology of IH without mapping linearly onto EDS severity.

### 4.3 Sleep Spindle Dynamics in a Hyperstable Thalamocortical System

The current results extend previous evidence of spindle abnormalities in central hypersomnolence disorders [19, 20]. IH participants exhibited reduced spindle amplitude, while spindle density remained comparable to controls. This dissociation between spindle quantity and morphology suggests that thalamocortical circuits can initiate oscillatory events but engage them with reduced synchrony. In healthy sleepers, robust spindle expression reflects effective recruitment of thalamocortical synchrony to protect stability against internal or environmental perturbations [18, 42]. In IH, however, N3 sleep appears particularly stable and potentially resistant to transitions toward lighter sleep stages or wakefulness. N2 sleep also showed reduced transitions toward wakefulness and N3, together with increased transitions to N1, indicating a boundary‐dependent reorganization of NREM dynamics rather than uniform NREM stabilization. This pattern may imply that the thalamocortical system does not need to engage as strongly in its protective function. Accordingly, spindle amplitude attenuation may reflect reduced reliance on thalamocortical synchronizing mechanisms, representing a secondary effect of sleep hyperstability rather than a deficit in spindle generation. Consistent with this interpretation, spindle amplitude correlated positively with mean sleep latency on the MSLT, suggesting that individuals with greater daytime sleepiness also showed weaker spindle expression. This relationship underscores the functional relevance of spindle morphology as an index of thalamocortical engagement.

### 4.4 Physiological Interpretation of Thalamocortical Over‐Inhibition

The co‐occurrence of attenuated spindles, unstable yet prolonged REM dynamics, and rigid NREM‐N3 patterns suggests that IH may involve a state of thalamocortical over‐inhibition. Excessive GABAergic tone within the thalamic reticular nucleus or its cortical projections could suppress oscillatory amplitude and slow transitions between vigilance states, yielding a network that maintains sleep continuity but exhibits limited adaptive modulation [43]. This view aligns with models proposing that sleep–wake regulation depends on a precise balance between inhibition and thalamocortical coupling [44]. When this balance shifts toward inhibition, neural networks enter a low‐gain regime—stable yet unresponsive to change.

Within this regime, NREM and REM systems may express complementary manifestations of the same inhibitory bias. In NREM sleep, thalamocortical over‐inhibition promotes excessive synchrony and persistence of deep NREM sleep, contributing to state inertia and impaired reactivation of ascending arousal systems [40]. In REM sleep, the same inhibitory overload may destabilize the flip‐flop switch between REM‐on and REM‐off populations. Rodent studies show that pharmacological inactivation of REM‐off GABAergic neurons in the ventrolateral periaqueductal gray matter increases REM duration but fragments its continuity, producing frequent transitions between REM and light NREM [45]. In IH, a similar overinhibited REM‐off system could sustain REM expression while preventing its consolidation, yielding oscillations between REM and N1 rather than transitions to wakefulness. From a systems perspective, these features converge with the framework of thalamocortical dysrhythmia [46], yielding sleep that is deep and continuous yet paradoxically non‐restorative, the electrophysiological hallmark of IH.

### 4.5 Limitations and Future Directions

The retrospective design limits causal inference, and a single‐night PSG may not fully capture night‐to‐night variability. The potential influence of hormonal contraceptive use and menstrual cycle phase could not be assessed in this retrospective cohort, and objective and subjective measures of daytime sleepiness were not available in healthy controls. In addition, several clinically relevant features of IH, including habitual sleep duration, sleep inertia/sleep drunkenness, and restorative daytime naps, were not systematically documented (but rather as descriptive clinical notes) in this retrospective cohort, precluding assessment of their associations with the observed sleep microstructural alterations. Future high‐density and longitudinal EEG studies should clarify whether spindle attenuation arises from an overinhibited thalamocortical network, reduced cortical responsiveness, or altered thalamocortical coupling. They should also determine how the sleep microstructural alterations identified here relate to the broader clinical phenotype of IH.

## 5 Conclusion and Clinical Implications

This study combined a validated spindle‐detection algorithm with data‐driven analyses of sleep‐state dynamics in a relatively large cohort, enabling a fine‐grained characterization of sleep regulation in IH. The findings delineate a distinctive electrophysiological profile marked by increased REM‐N1 fragmentation together with increased stability at the REM‐wake boundary, excessive stabilization of N3 sleep, and reduced spindle amplitude despite preserved density, consistent with rigid sleep–wake boundaries and reduced adaptability. These features contrast with the state instability typical of narcolepsy, offering potential biomarkers to refine diagnosis and inform interventions aimed at restoring adaptive state regulation and improving daytime alertness. By moving beyond standard sleep‐architecture metrics, this work offers novel insight into the neurophysiological basis of IH and underscores the need for mechanistic markers that capture sleep dynamics and inform targeted treatments to restore adaptive state regulation.

## Author Contributions

All authors have seen and approved this manuscript. Samantha Mombelli participated in the design of the investigation, completed statistical analyses, and served in a primary role in manuscript construction. Nadia Gosselin participated in the design of the investigation, guided statistical analyses, and served in a primary role in manuscript construction. Karine Lacourse, Boshra Khajehpiri, and Jean Marc Lina supported and reviewed the statistical analyses. Anne‐Sophie Deshaies‐Rugama, Hélène Blais, and Cynthia Thompson acted in primary roles for data management, while also contributing to the development of the manuscript. Alex Desautels, Jacques Montplaisir, Milan Nigam, Christophe Moderie, Catherine Duclos, and Julie Carrier participated in the data collection, while also providing a critical review of the manuscript.

## Funding

This work was supported by the American Academy of Sleep Medicine Foundation, Grant 227‐SR‐20.

## Conflicts of Interest

Nadia Gosselin has received honoraria for Eisai and sponsorships for Jazz Pharmaceuticals, Eisai, Axsome, and Paladin, but these contributions are not related to the present study. Julie Carrier currently benefits from an Eisai grant, but these contributions are not related to this study. Alex Desautels received research grants from Eisai and Takeda; honoraria from serving on the scientific advisory board of Eisai, Takeda, Paladin Labs, as well as honoraria from speaking engagements from AstraZeneca, Eisai, Jazz Pharma, and Paladin Labs. None of the financial disclosures is relevant to the submitted work. Other authors report no conflicts of interest related to this article.

## Supporting information


**S1.** Principal component analysis.
**Figure 1‐S1**. Scree plot.
**Table 1‐S1**. Component characteristics.
**S2**. Sleep bout duration and sleep stage transition variables.
**Table 1‐S2**. Sleep bout duration of IH and control groups, adjusted for age and sex.
**Table 2‐S2**. Sleep stage transition index of IH and control groups, adjusted for age and sex.
**S3**. Analysis of spindle‐related variables during N2 sleep stages.
**Table 1‐S3**. All‐night spindle characteristics detected during N2 at central derivations (C3–C4) in IH and healthy controls, adjusted for age and sex using the SUMO algorithm.

## Data Availability

Relevant data that support the findings of this study are available from the corresponding author upon reasonable request.
