# Supplementary material for: Boundary‐Dependent Sleep–Wake Dysregulation in Idiopathic Hypersomnia
Source: Ann Clin Transl Neurol. 2026 Sep 2:10.1002/acn3.70522. Online ahead of print. doi: 10.1002/acn3.70522 (PMC13538280; doi:10.1002/acn3.70522)
Supplement: Supplementary file 1 [file ACN3-9999-0-s001.docx]

**Boundary-dependent sleep-wake dysregulation in idiopathic hypersomnia**

Samantha Mombelli^1,2^, Karine Lacourse^1^, Hélène Blais^1^, Anne-Sophie Deshaies-Rugama^1,3^, Cynthia Thompson^1^, Alex Desautels^1,4^, Jacques Montplaisir^1,2^, Milan Nigam^1,4^, Christophe Moderie^1,2^, Boshra Khajehpiri^1,5,6^, Catherine Duclos^1,7^, Jean Marc Lina^1,5,6^, Julie Carrier^1,3^, Nadia Gosselin^1,3^

**Author affiliations:**

1 Center for Advanced Research in Sleep Medicine, Research center of the Centre intégré universitaire de santé et de services sociaux du Nord de l’Île-de-Montréal, Montreal, Canada

2 Department of Psychiatry and Addictology, Université de Montréal, Montreal, Canada

3 Department of Psychology, Université de Montréal, Montreal, Canada

4 Department of Neuroscience, Université de Montréal, Montreal, Canada

5 LIVIA, ILLS, École de technologie supérieure (ÉTS), Université du Québec, Montreal, Canada

6 Department of Electrical Engineering, École de Technologie Supérieure, Montreal, Quebec, Canada

7 Département d'anesthésiologie et de médecine de la douleur, Université de Montréal, Montréal, Canada

Correspondence to:

Nadia Gosselin, Ph.D.

Center for Advanced Research in Sleep Medicine

Hôpital du Sacré-Cœur de Montréal

5400 boul. Gouin Ouest, office J-5135, Montréal, Québec, H4J 1C5, Canada

Tel: 514-338-2222 ext. 7717; Fax: 514-338-3893

Email: [nadia.gosselin@umontreal.ca](mailto:nadia.gosselin@umontreal.ca)

### S1. SUPPLEMENTARY MATERIALS: Principal Component Analysis:

### Figure 1-S1 Scree Plot

### The scree plot and the component summary table illustrate the results of the principal component analysis performed on selected sleep bout and transition variables.

| **Table 1-S1** Component Characteristics | | | | | | | | | | | | | | | |
| --- | --- | --- | --- | --- | --- | --- | --- | --- | --- | --- | --- | --- | --- | --- | --- |
|  |  | | Unrotated solution | | | | | | Rotated solution | | | | | |  |
|  |  | | Eigenvalue | | Proportion var. | | Cumulative | | SumSq. Loadings | | Proportion var. | | Cumulative | |  |
|  | Component 1 |  | 6.190 |  | 0.229 |  | 0.229 |  | 5.997 |  | 0.222 |  | 0.222 |  |  |
|  | Component 2 |  | 4.689 |  | 0.174 |  | 0.403 |  | 4.477 |  | 0.166 |  | 0.388 |  |  |
|  | Component 3 |  | 3.537 |  | 0.131 |  | 0.534 |  | 3.236 |  | 0.120 |  | 0.508 |  |  |
|  | Component 4 |  | 1.900 |  | 0.070 |  | 0.604 |  | 2.395 |  | 0.089 |  | 0.596 |  |  |
|  | Component 5 |  | 1.665 |  | 0.062 |  | 0.666 |  | 1.876 |  | 0.069 |  | 0.666 |  |  |
|  |  | | | | | | | | | | | | | |  |

### S2. SUPPLEMENTARY MATERIALS: sleep bout duration and sleep stage transition variables

### Table 1-S2 reports the results of the sleep bout duration analyses. The IH group exhibited significantly longer REM sleep bouts compared to controls, specifically from the 4th to the 10th longest uninterrupted REM episodes. In contrast, no significant group differences were found for N2-N3 bout durations.

**Table 1-S2 Sleep bout duration of IH and control groups, adjusted for age and sex**

|  | | **IH n=62** | **Controls n=81** | | ***F* values**  **(1, 139)** | | **P-value*** | **Effect sizes** |
| --- | --- | --- | --- | --- | --- | --- | --- | --- |
| **N2-N3 sleep bouts, min** |  | | |  | |  | | |
| 1^st^ bout | | 54.87 ± 14.95 | 49.71 ± 13.62 | | 4.238 | | 0.242 | 0.025 |
| 2^nd^ bout | | 44.07 ± 10.00 | 40.64 ± 10.12 | | 3.827 | | 0.052 | 0.022 |
| 3^rd^ bout | | 36.23 ± 9.52 | 33.41 ± 8.83 | | 2.512 | | 0.115 | 0.015 |
| 4^th^ bout | | 28.73 ± 8.02 | 27.52 ± 7.15 | | 0.342 | | 0.559 | 0.002 |
| 5^th^ bout | | 22.76 ± 6.90 | 22.10 ± 6.79 | | 0.097 | | 0.755 | 6.4453x10^-4^ |
| 6^th^ bout | | 18.19 ± 5.48 | 17.80 ± 4.96 | | 0.034 | | 0.853 | 2.357x10^-4^ |
| 7^th^ bout | | 14.68 ± 4.95 | 14.62 ± 4.43 | | 0.030 | | 0.862 | 2.096x10^-4^ |
| 8^th^ bout | | 11.90 ± 4.04 | 12.16 ± 3.93 | | 0.232 | | 0.631 | 0.002 |
| 9^th^ bout | | 9.81 ± 3.15 | 10.15 ± 3.69 | | 0.473 | | 0.493 | 0.003 |
| 10^th^ bout | | 8.74 ± 2.80 | 8.43 ± 3.08 | | 0.259 | | 0.612 | 0.002 |
| Mean of 10 longest N2-N3 bouts | | 25.02 ± 4.94 | 23.65 ± 4.51 | | 2.279 | | 0.133 | 0.013 |
| SD of 10 longest N2-N3 bouts | | 15.35 ± 4.72 | 13.67 ± 4.63 | | 4.058 | | 0.242 | 0.025 |
| Mean of all uninterrupted N2-N3 bouts | | 9.27 ± 3.66 | 9.72 ± 4.75 | | 1.150 | | 0.285 | 0.007 |
| SD of all uninterrupted N2-N3 bouts | | 13.42 ± 4.17 | 12.88 ± 4.27 | | 0.169 | | 0.682 | 0.001 |
| **REM sleep bouts, min** | |  |  | |  | |  |  |
| 1^st^ bout | | 18.48 ± 6.82 | 19.98 ± 7.94 | | 1.900 | | 0.170 | 0.013 |
| 2^nd^ bout | | 13.96 ± 4.63 | 13.40 ± 4.81 | | 0.282 | | 0.596 | 0.002 |
| 3^rd^ bout | | 10.94 ± 3.91 | 9.86 ± 3.67 | | 2.341 | | 0.128 | 0.016 |
| 4^th^ bout | | 8.86 ± 3.10 | 7.63 ± 3.05 | | 5.403 | | **0.044** | 0.037 |
| 5^th^ bout | | 7.43 ± 2.64 | 6.31 ± 2.52 | | 6.640 | | **0.025** | 0.045 |
| 6^th^ bout | | 6.07 ± 2.27 | 4.87 ± 2.23 | | 10.508 | | **0.004** | 0.069 |
| 7^th^ bout | | 5.09 ± 2.10 | 4.07 ± 2.02 | | 8.582 | | **0.011** | 0.060 |
| 8^th^ bout | | 4.48 ± 2.17 | 3.39 ± 1.74 | | 10.916 | | **0.004** | 0.076 |
| 9^th^ bout | | 3.82 ± 1.81 | 2.79 ± 1.60 | | 12.901 | | **0.004** | 0.092 |
| 10^th^ bout | | 3.28 ± 1.44 | 2.47 ± 1.46 | | 9.116 | | **0.010** | 0.074 |
| Mean of 10 longest REM bouts | | 8.38 ± 2.43 | 7.85 ± 2.30 | | 1.296 | | 0.257 | 0.009 |
| SD of 10 longest REM bouts | | 4.90 ± 2.17 | 5.66 ± 2.67 | | 4.140 | | 0.068 | 0.028 |
| Mean of all uninterrupted REM bouts | | 5.97 ± 2.78 | 6.36 ± 2.70 | | 1.073 | | 0.302 | 0.007 |
| SD of all uninterrupted REM bouts | | 5.20 ± 2.07 | 6.01 ± 2.70 | | 4.568 | | 0.059 | 0.031 |

Sleep bouts are ordered by duration, with the longest bout listed first. Data are presented as mean ± standard deviation. *P-values are corrected for multiple comparisons.

Legend: SD indicates the standard deviation.

Table 2-S2 reports the results of sleep stage transition analyses. The IH group exhibited significantly lower transition index from wake to N1 and N2 sleep. They also showed a reduced index of transitions from N2 to wake and to N3, as well as from N3 to wake and N2 sleep. Conversely, the IH group demonstrated a significantly higher index of transitions from N2 to N1, from N1 to REM sleep, and from REM sleep to N1.

**Table 2-S2 Sleep stage transition index of IH and control groups, adjusted for age and sex**

|  | | **IH n=62** | **Controls n=81** | | ***F* values**  **(1, 139)** | | **P-value*** | **Effect sizes** |
| --- | --- | --- | --- | --- | --- | --- | --- | --- |
| Total sleep stage transition index, n/h | | 22.04 ± 6.81 | 24.21 ± 6.92 | | 2.801 | | 0.096 | 0.016 |
| **Sleep transition index from wake** |  | | |  | |  | | |
| Wake to N1, n/h | | 2.33 ± 1.14 | 3.19 ± 1.67 | | 10.526 | | **0.004** | 0.063 |
| Wake to N2, n/h | | 0.36 ± 0.31 | 0.56 ± 0.46 | | 8.483 | | **0.008** | 0.055 |
| Wake to N3, n/h | | 0.004 ± 0.03 | 0.016 ± 0.06 | | 2.612 | | 0.108 | 0.018 |
| Wake to REM, n/h | | 0.27 ± 0.33 | 0.26 ± 0.30 | | 0.019 | | 0.890 | 1.327x10^-4^ |
| **Sleep transition index from N1** | |  |  | |  | |  |  |
| N1 to wake, n/h | | 0.86 ± 0.66 | 1.22 ± 1.11 | | 3.921 | | 0.050 | 0.026 |
| N1 to N2, n/h | | 4.35 ± 2.26 | 4.06 ± 1.92 | | 2.045 | | 0.115 | 0.012 |
| N1 to N3, n/h | | 0.019 ± 0.05 | 0.028 ± 0.11 | | 0.424 | | 0.516 | 0.003 |
| N1-REM, n/h | | 1.09 ± 0.74 | 0.81 ± 0.64 | | 7.459 | | **0.028** | 0.045 |
| **Sleep transition index from N2** | |  |  | |  | |  |  |
| N2 to wake, n/h | | 1.22 ± 0.64 | 1.78 ± 1.00 | | 12.870 | | **0.004** | 0.083 |
| N2 to N1, n/h | | 2.73 ± 1.99 | 2.00 ± 1.44 | | 10.109 | | **0.004** | 0.059 |
| N2 to N3, n/h | | 2.69 ± 1.61 | 3.54 ± 1.93 | | 8.852 | | **0.004** | 0.058 |
| N2 to REM, n/h | | 1.02 ± 0.52 | 1.04 ± 0.57 | | 0.071 | | 0.791 | 3.439x10^-4^ |
| **Sleep transition index from N3** | |  |  | |  | |  |  |
| N3 to wake, n/h | | 0.16 ± 0.19 | 0.26 ± 0.27 | | 6.471 | | **0.024** | 0.043 |
| N3 to N1, n/h | | 0.071 ± 0.11 | 0.055 ± 0.14 | | 0.480 | | 0.490 | 0.003 |
| N3 to N2, n/h | | 2.46 ± 1.51 | 3.24 ± 1.86 | | 8.580 | | **0.016** | 0.056 |
| N3 to REM, n/h | | 0.023 ± 0.06 | 0.023 ± 0.05 | | 0.002 | | 0.962 | 1.655x10^-4^ |
| **Sleep transition index from REM** | |  |  | |  | |  |  |
| REM to wake, n/h | | 0.60 ± 0.44 | 0.68 ± 0.51 | | 0.705 | | 0.403 | 0.004 |
| REM to N1, n/h | | 1.21 ± 0.74 | 0.88 ± 0.63 | | 9.704 | | **0.008** | 0.059 |
| REM to N2, n/h | | 0.56 ± 0.51 | 0.55 ± 0.55 | | 1.417x10^-4^ | | 0.982 | 3.496x10^-6^ |
| REM to N3, n/h | | 0.00 ± 0.00 | 0.002 ± 0.017 | | 0.991 | | 0.321 | 0.007 |

Data are presented as mean ± standard deviation. *P-values are corrected for multiple comparisons.

The transitions indices for each variable were calculate as the number of sleep stage transitions/hour

S3. SUPPLEMENTARY MATERIALS: **Analysis of spindle-related variables during N2 sleep stages**

**Table 1-S3** All-night spindle characteristics detected during N2 at central derivations (C3–C4) in IH and healthy controls, adjusted for age and sex using the SUMO algorithm.

|  | IH  (n = 62) | Controls  (n = 81) | F values  (1, 139) | p-values* | Effect sizes |
| --- | --- | --- | --- | --- | --- |
| Density, n/min | 3.54 ± 2.06 | 3.85 ± 2.12 | 1.013 | 0.316 | 0.007 |
| Duration, sec | 0.85 ± 0.10 | 0.87 ± 0.10 | 1.420 | 0.235 | 0.008 |
| Spindle average frequency (Hz) counting peaks | 13.52 ± 0.43 | 13.36 ± 0.43 | 3.012 | 0.085 | 0.018 |
| Peak-to-peak amplitude, µV | 31.69 ± 6.71 | 33.41 ± 6.35 | 6.763 | **0.025** | 0.035 |
| rms (Root Mean Square) amplitude, µV | 8.16 ± 1.68 | 8.66 ± 1.61 | 7.829 | **0.025** | 0.041 |

*P-values are corrected for multiple comparisons.

**Partial correlation adjusted for sex, and age:**

No correlations were found between ESS scores and spindles characteristics in IH patients.

However, more severe EDS as measured with shorter MSLT latencies, correlated with some anomalies in spindles characteristics in IH participants. More specifically, shorter MSLT were significantly correlated with smaller peak to peak amplitude (r = 0.292, p = 0.024) and, smaller RMS amplitude (r = 0.309, p = 0.016)
